# The effects of eccentric training on hamstring flexibility and strength in young dance students

**DOI:** 10.1038/s41598-024-53987-0

**Published:** 2024-02-14

**Authors:** Feng Liang, Huo Hongfeng, Zhu Ying

**Affiliations:** 1 School of Physical Education, Hebei Normal University, No. 20, East South Second Ring Road, Shijiazhuang, 050024 Hebei China; 2 Art and Sports College, HeBei institute of communications, No. 8, Police Road, Xinhua District, Shijiazhuang, 051430 Shijiazhuang, China; 3 Department of Dance, School of Music, Hebei Normal University, No. 20, South Second Ring Road, Shijiazhuang, 050024 Hebei China

**Keywords:** Eccentric training, Hamstring, Flexibility, Strength, Dance, Musculoskeletal system, Bone quality and biomechanics

## Abstract

The objective of this research is to examine the impact of eccentric training on hamstring flexibility and strength in young dancers during the concluding stages of their foundational dance training program. A total of 24 female, second-year dance students from Hebei Normal University were selected as participants. They were divided into three distinct groups: Nordic hamstring exercise and single-leg deadlift group (NHE&SLD), forward bending exercises and standing leg lift group (FBE&SLL), and a control group (CG). The study was designed around a 6-week training regimen. An isokinetic dynamometer was used to measure seated knee flexor–extensor strength, while electronic goniometry was employed to measure hamstring flexibility in the supine position. Paired sample *t*-tests were conducted within each group, and one-way analysis of covariance (ANCOVA) was utilized for comparisons between groups. In the NHE&SLD group, significant disparities were observed in both concentric (T = − 5.687, P = 0.001) and eccentric (T = − 3.626, P = 0.008) hamstring strength pre and post-intervention. The pre-intervention dominant leg concentric strength test values significantly influenced the post-intervention outcomes (F = 5.313, P = 0.001, η^2^ = 0.840). Similarly, the pre-intervention dominant leg eccentric strength test values impacted the post-intervention results (F = 4.689, P = 0.043, η^2^ = 0.190). Following the intervention, the NHE&SLD group displayed marked changes in the active straight leg raising angle on both left (T = − 4.171, P = 0.004) and right (T = − 6.328, P = 0.001) sides. The FBE&SLL group also revealed significant changes in the active straight leg raising angle on both left (T = − 4.506, P = 0.003) and right (T = − 4.633, P = 0.002) sides following the intervention. The pre-intervention left leg concentric strength test value significantly influenced the post-intervention outcomes (F = 25.067, P = 0.001, η^2^ = 0.556). Likewise, the pre-intervention right leg eccentric strength test value significantly influenced the post-intervention results (F = 85.338, P = 0.01, η^2^ = 0.810). Eccentric training can better enhance the flexibility and strength of hamstring muscles in dance students. Traditional stretching training significantly improves the flexibility of the hamstring muscles. Eccentric training has more training benefits than traditional stretching training. It is recommended for dance students to use eccentric training when increasing hamstring flexibility and strength.

## Introduction

In the process of dance performance and training, the comprehensive and profound development and exploration of bodily expression are the objectives^1^. To achieve these, dancers need to possess a comprehensive set of physical skills, including flexibility, strength, explosive effort and stability, among others. Flexibility and strength are foundational for enhancing all other physical abilities^2,3^. However, there is a certain contradiction between the two, making it challenging to strike a balance. Excessive flexibility can lead to a decrease in strength and vice versa. The question of how to improve the flexibility and strength of dance students, maintain body aesthetics, and also have training methods that are easy to implement and time-efficient, has always been a significant concern in dance education.

Stretching is considered an important method for warming up, promoting physical health, and developing flexibility^4,5^. However, over the past 20 years, there has been some controversy regarding the use of stretching as a warm-up or regular training routine (i.e., stretching over weeks) to improve joint mobility, enhance performance, and promote health. Some reports suggest that using static stretching as a pre-activity strategy can lead to decreased physical performance (such as strength, agility, speed, balance)^6,7^. The effectiveness of dynamic and static stretching in improving joint mobility is largely undisputed, but stretching training may not be the only technique to improve range of motion (ROM). Eccentric training is considered a potent method for muscle development and increasing strength, compared to isometric and concentric contractions. Eccentric training can promote greater neuromuscular activation capacity, and similar to static stretching, it is effective in improving hamstring flexibility. Related research indicates that eccentric hamstring exercises can extend the length of the biceps femoris muscle fibers, albeit without a significant alteration in muscle thickness^8–10^. Drawing from this evidence and the research needs of the present study, we aim to examine the effects of eccentric hamstring training during the concluding part of a foundational dance training course on the hamstring flexibility and muscle strength of young dance students. We seek to obtain valuable outcomes from this study, which can lay the groundwork for dance education, training, and related research, and provide theoretical and technical support. Hypothesis: hamstring training provides a better increase in hamstring muscle flexibility and muscle strength than traditional stretching training.

### Research participants and methods

#### Research participants

This study focuses on the hamstring flexibility and muscle strength of 24 s-year female students from the Dance Department at Hebei Normal University. Sample size calculations were performed through GPower 3.1.9. With 3 groups (NHE&SLD, FBE&SLL, and CG), 2 tests, statistical efficacy of 0.8, α = 0.05, β = 0.2, and statistical method of one-way analysis of covariance (ANCOVA), the final calculations were obtained, and the minimum sample size for the present study was 8 persons per group. Considering the fact that variables such as training content and intensity can vary if the subject population comes from different grades, which can affect the results of the intervention, thus insisting on a class-based approach, the minimum sample size of 8 persons per group was used. To eliminate potential biases from gender differences and the impact of regular training on research outcomes, only female students from the same class were selected as research participants. To avoid subjective selection bias, the 24 participants were randomly allocated to three groups, each comprising 8 participants (n = 8). Group 1 participated in Nordic hamstring exercises and single-leg deadlifts (NHE&SLD), Group 2 conducted forward bend exercises and standing leg lifts (FBE&SLL), and Group 3 functioned as the control group (CG). As depicted in Table 1, all research participants possessed over 4 years of professional dance training experience. There were no significant differences in the groups for parameters such as age, weight and height (P > 0.05).They had no joint or muscle injuries within the past 6 months and had not undergone any surgeries within the past year. Prior to the intervention, the participants were informed of the study’s purpose and content. All participants voluntarily agreed to partake in the experiment and signed informed consent forms. This study was approved by the Biomedical Ethics Committee of Hebei Normal University (2022llsc026).

**Table 1:** Basic information of the study subjects (n = number, M ± SD).

Group	n	Age (years)	Stature (cm)	Body mass (kg)
NHE&SLD	8	19.63 (1.0)	164.75 (4.9)	53.74 (5.0)
FBE&SLL	8	19.23 (0.7)	166.11 (6.9)	56.24 (7.0)
CG	8	18.93 (0.6)	166.25 (3.8)	55.84 (8.1)

### Intervention experimental design

#### Eccentric intervention methods

**Figure 1:**
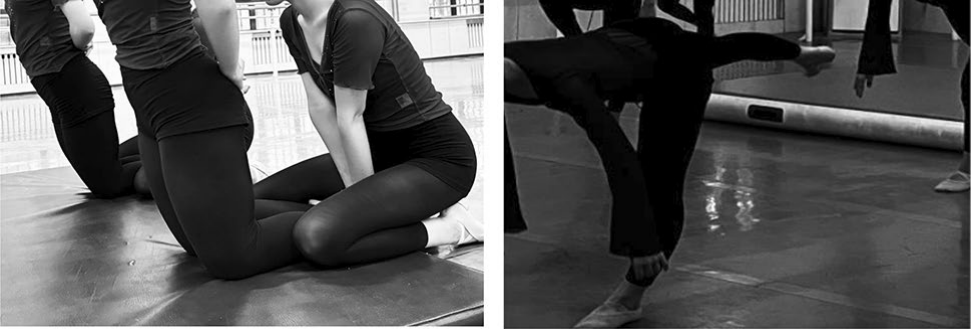
Centrifugal intervention means.

Nordic hamstring exercises^11^ (Fig. 1): This is an effective regimen for training the eccentric contraction capacity of the hamstring muscles, requiring the assistance of another person. The participant kneels with legs together and hands crossed in front of the chest, while the assistant stabilizes the legs. During the exercise, the trainer fully extends the hips, maintains proper alignment between the trunk and lower limbs, and leans the body forward to overcome gravity, inducing an eccentric contraction of the hamstring muscles. Throughout the process, the trainer aims to maintain trunk stability and prolongs the time of inclination until the hands touch the ground, then uses both arms to push off the ground and return to the starting position.

Single-leg deadlift exercise^12^ (Fig. 1): This is also an effective regimen for training the eccentric contraction capacity of the hamstring muscles. In comparison to Nordic exercises, it can also enhance lower limb dynamic stability and posture control^13^. The participant stands with a slightly bent supporting leg, tightens the trunk, and prepares the moving leg for hip and knee flexion at 90°. During the exercise, the moving leg is extended backward to a horizontal position with the ground, while the trunk is flexed forward to be parallel to the ground, and the arms hang naturally. Throughout the process, the participant maintains trunk stability, actively flexes the hip, and precisely activates the hamstring muscles of the supporting leg. After the fingertips touch the ground, the participant extends the hip to return to the initial position.

#### Traditional stretching methods

**Figure 2:**
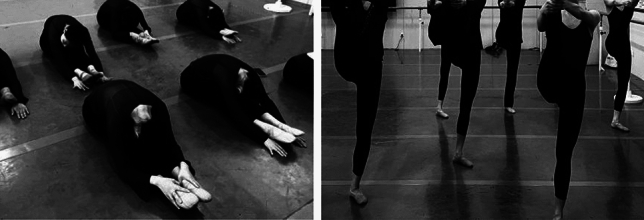
Traditional stretching means.

Seated forward bend exercise (Fig. 2): Begin in a seated position with legs together and arms resting at the sides. Exhale, tuck the chin, extend the spine, rotate the pelvis, and perform the maximum hip flexion forward bend. The stretch is controlled at the position where the stretching sensation is most pronounced. Throughout the process, try to increase the range of forward bend by adjusting the depth of breath.

Standing leg lift exercise (Fig. 2): Stand on one foot, fully extend the supporting leg, maintain a neutral pelvis, and lift the active leg upward. Both hands are used to hold onto the ankle of the active leg. During this process, ensure a neutral pelvis, fully stretch the posterior muscles of the active leg, and extend the supporting leg.

#### Implementation of intervention plan

The experimental process comprises a pre-test, a 6-week eccentric training intervention, and a post-test. The interventions were executed following the completion of basic dance training classes. The Nordic hamstring exercise and single-leg deadlift (NHE&SLD) group performed Nordic hamstring exercises initially, took a 3-min rest, and then conducted single-leg deadlift exercises. The forward bend exercise and standing leg lift (FBE&SLL) group firstly conducted seated forward bend exercises, followed by a 3-min rest, and then performed standing leg lift exercises. The third group served as the control group (CG). The training content is set up for according to the relevant research experience, and the specific content is shown in Table 2. The rest time between the intervention groups was all at 30 s. Participants came from the same class, and only the intervention programs after randomization were different, while other daily training volume and level of living activities were similar.

**Table 2:** The 6-week training program between NHE & SLD and FBE & SLL groups.

Week	Every week (time)	Group count × frequency	Week	Every week (time)	Group count × time
1–2 Weeks	2	3 × 6–8	1–2 Weeks	2	3 × 30 s
3–4 Weeks	2	3 × 12, 10, 8	3–4 Weeks	2	3 × 45 s
5–6 Weeks	2	3 × 12	5–6 Weeks	2	3 × 60 s

### Experimental testing content

#### Isokinetic muscle strength testing

**Figure 3:**
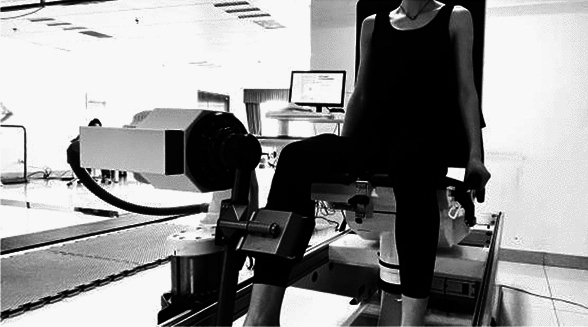
An isokinetic muscle strength test.

Isokinetic muscle strength testing^14^: We employed the Physiomed Elektromedizin AG isokinetic dynamometer (Model CON-TREY PM-2200301 [50/60]Hz, MADE IN GERMANY) to assess knee joint muscle strength in the dominant leg of young dancers, ascertained through the kicking test (Fig. 3). The contraction modes for the knee joints included concentric-concentric and eccentric-eccentric contractions. The testing speed was set at 60°/s, allowing for the evaluation of the muscle strength level during knee joint flexion and extension; the testing action involved ongoing flexion and extension movements comprising 6 repetitions per set. The knee joint muscle strength testing was conducted with the participant seated, within a range of motion from 5° to 85°. The assessed parameter was the Relative Peak Torque (RPT).

#### Active straight leg raise test

The Active Straight Leg Raise (ASLR) test^15^, a hip flexion range of motion assessment, is recognized as a reliable and valid test within the Functional Movement Screen (FMS). The ASLR test measures the active flexibility of hip flexors and reflects core stability as well as the stability of the contralateral hip joint against movement resistance. It further evaluates the flexibility of hamstring and calf muscles, alongside the stability provided by iliopsoas and lumbar muscles in pelvis stabilization.

During the test, the participant lies supine with one knee positioned posteriorly on an FMS testing board and the foot's toes pointing vertically upward. The other leg performs an active straight leg raise. Both legs align straight at the 0-degree marker on a goniometer, with the goniometer's center placed at the femoral greater trochanter. The goniometer's proximal end aligns along the body's horizontal line while the distal end is positioned at the femur's lateral malleolus. The leg raise halts if the opposite foot's toes rotate either inwards or outwards or if the knee lifts off the FMS testing board. The test is carried out twice on each side, and the highest value is documented in degrees rounded to the nearest whole number. If any pain is experienced during the test, the measurement is discontinued.

#### Experimental testing schedule

The pre-test was performed a week prior to the intervention training whereas the post-test was conducted in the week immediately following the end of intervention training. Participants were advised to avoid strenuous activities within a 48-h timeframe leading up to each test, and were directed to maintain their usual lifestyle patterns and normal dietary intake within the 72 h prior to testing. To counteract any influence of timing, all tests took place at the same time of day. Before starting the tests, a standardized warm-up was executed, comprising 5 min of jogging and 5 min of dynamic stretching. All methods were performed in accordance with the relevant guidelines and regulations.

### Data statistical analysis

Data analysis was carried out utilizing SPSS 24 statistical package. Test results were presented as mean ± standard deviation (M ± SD). Paired sample *t*-tests were employed for intra-group comparisons. Given the disparate testing items, paired sample *t*-tests were utilized within groups, while a one-way analysis of covariance (ANCOVA) was utilized between groups. The level for statistical significance was fixed at P < 0.05.

## Research results

### Isokinetic muscle strength test results

The K–S test was used to assess normal distribution of pre-test and post-test data for isokinetic muscle strength within groups (P > 0.05). The results of the paired sample *t*-tests are displayed in Table 3. The NHE&SLD group demonstrated significant differences in the strength levels of the dominant leg for concentric contraction (T = − 5.687, P = 0.001) and for eccentric contraction (T = − 3.626, P = 0.008) before and after the intervention training. The FBE&SLL group and the CG group did not show significant differences in the strength levels of the dominant leg for concentric (FBE&SLL: T = − 2.283, P = 0.056; CG: T = − 1.260, P = 0.078) and eccentric contraction (FBE&SLL: T = − 2.038, P = 0.081; CG: T = − 2.257, P = 0.059) before and after the intervention training.

**Table 3:** Comparison of intra-and post-test between concentric and eccentric muscle strength groups of the dominant legs (unit: Nm).

Group	Intra-concentric	Post-concentric	*P*	Intra-eccentric	Post-eccentric	*P*
NHE&SLD	59.20 (7.6)	69.46 (7.5)	0.001	91.97 (5.0)	130.35 (13.5)	0.008
FBE&SLL	54.88 (5.0)	57.92 (6.8)	0.056	86.91 (3.7)	104.22 (6.9)	0.081
CG	55.38 (6.2)	62.10 (11.0)	0.078	93.28 (5.2)	96.16 (9.2)	0.059

Due to the disparity in initial muscle strength levels of the participants before the intervention, individuals with higher pre-test scores exhibited relatively less potential for improvement. In fact, these pre-test scores exerted influence on the post-test scores. It would therefore be unjustified to merely consider the difference between post-test and pre-test scores, without accounting for the pre-test scores. Thus, a one-way ANCOVA was chosen with the pre-test data serving as the covariate, to evaluate the impact of different intervention methods on lower extremity flexibility.

Levene's test for homogeneity of variance confirmed equal variances among the residuals of the dependent variable across the groups (F = 3.47, P = 0.03). Scatter plots were utilized to illustrate the linear relationship between the pre- and post-test data within each group, satisfying the requirements for one-way ANCOVA. Following the removal of the influence of the pre-test data (covariate) on the post-test data and the formation of residuals, a variance analysis of these residuals was conducted, producing the results displayed in Table 4. According to the one-way ANCOVA outcomes, the pre-intervention concentric muscle strength test value of the dominant leg, when employed as a covariate, held significant influence over the degree of change in post-intervention outcomes (F = 5.313, P = 0.001, η^2^ = 0.840). Likewise, the pre-intervention eccentric muscle strength test value of the dominant leg, utilized as a covariate, significantly affected the degree of change in post-intervention outcomes (F = 4.689, P = 0.043, η^2^ = 0.190). Specifically, in comparison to the FBE&SLL group and the CG group, the NHE&SLD group exhibited significant differences in post-intervention concentric muscle strength levels (P = 0.001) and eccentric muscle strength levels (P = 0.008). The gains in concentric and eccentric muscle strength levels after the intervention in the FBE&SLL group (P = 0.100 and P = 1.000, respectively) and in the CG group (P = 0.148 and P = 1.000, respectively) did not reach statistical significance. These findings suggest that, in regard to intergroup comparisons, the NHE&SLD group demonstrated the most substantial improvements in both concentric and eccentric muscle strength levels. The FBE&SLL and CG groups did not significantly increase the centripetal and centrifugal systolic muscle strength level after the intervention.

**Table 4:** Results of centrifugal muscle strength after unadjusted and adjusted for covariates (unit: Nm).

Group		Unadjusted		After adjustment		Group		Unadjusted		After adjustment	
		Average value	Standard deviation	Average value	Standard error			Average value	Standard deviation	Average value	Standard error
NHE&SLD	Concentric contraction	69.46	7.50	67.14*	1.27	NHE&SLD	Eccentric contraction	130.35	13.50	131.95^#^	6.62
FBE&SLL	Concentric contraction	57.92	6.87	57.31	1.26	FBE&SLL	Eccentric contraction	104.22	6.96	102.02	6.66
CG	Concentric contraction	62.10	11.02	63.02	1.25	CG	Eccentric contraction	96.16	9.2	96.75	6.59

### Active straight leg raise test results

Based on the results of the K–S test, the pre-test and post-test data within each group were determined to adhere to a normal distribution (P > 0.05). The results of the paired samples *t*-tests can be found in Table 5. Notably, significant differences were observed in the angle changes of active straight leg raise within the NHE&SLD group before and after the intervention, both on the left (T = − 4.171, P = 0.004) and right sides (T = − 6.328, P = 0.001). Likewise, significant disparities were observed in the FBE&SLL group in the angle changes of active straight leg raise before and after intervention, both on the left (T = − 4.506, P = 0.003) and right sides (T = − 4.633, P = 0.002). However, no significant differences were detected within the CG group in terms of the angle changes of active straight leg raise pre- and post-intervention, both on the left (T = − 1.859, P = 0.105) and right sides (T = − 1.328, P = 0.098). After the intervention, the NHE&SLD and FBE&SLL groups showed greater enhancements in the angle of active straight leg raise, whilst the CG group exhibited smaller changes.

**Table 5:** Comparison of pre-test and post-test between active straight knee leg lift group.

Group	Left front test (/°)	Left posterior test (/°)	*P*	Right front test (/°)	Right posterior test (/°)	*P*
NHE&SLD	100.46 (11.9)	112.87 (6.9)	0.004	105.26 (6.3)	118.60 (6.7)	0.001
FBE&SLL	102.85 (7.0)	114.71 ± (10.1)	0.011	105.91 (9.3)	113.88 (10.7)	0.002
CG	103.73 (10.1)	107.23 (12.6)	0.105	99.25 (11.9)	104.13 (5.3)	0.098

Levene's test for homogeneity of variances was performed, confirming that the residuals of the dependent variables between groups were characterized by equal variances. Scatter plots were created to demonstrate a linear relationship between the pre-test and post-test data within each group, thereby meeting the requirements for one-way ANCOVA analysis. As displayed in Table 6, according to the one-way ANCOVA analysis, the pre-intervention concentric muscle strength test values of the left leg when used as a covariate significantly loaded on the variation in the post-intervention results (F = 25.067, P = 0.001, η^2^ = 0.556). Likewise, pre-intervention eccentric muscle strength test values of the right leg used as a covariate significantly influenced the extent of the post-intervention results (F = 85.338, P = 0.01, η^2^ = 0.810). In comparison to the adjusted CG group, both the NHE&SLD group (P = 0.004) and the FBE&SLL group (P = 0.011) demonstrated significant differences in the left leg's angle changes during the active straight leg raise. Likewise, when compared to the adjusted CG group, both the NHE&SLD group (P = 0.001) and the FBE&SLL group (P = 0.002) displayed significant differences in the right leg's angle changes during the active straight leg raise. These findings suggest that, in terms of intergroup comparisons, both the NHE&SLD group and the FBE&SLL group evidenced the most considerable improvements in the active straight leg raise test for both legs, In the CG group, the active Angle of knee lift was less.

**Table 6:** Unadjusted and covariate adjusted results of active knee lift.

Group		Unadjusted (/°)		After adjustment (/°)		Group		Unadjusted (/°)		After adjustment (/°)	
		Average value	Standard deviation	Average value	Standard error			Average value	Standard deviation	Average value	Standard error
NHE&SLD	Left side	112.87	6.97	114.31*	2.47	NHE&SLD	Right side	118.60	6.78	116.97^#^	1.61
FBE&SLL	Left side	114.71	10.16	114.33^Δ^	2.45	FBE&SLL	Right side	113.88	10.57	111.64^※^	1.61
CG	Left side	107.23	12.61	106.17	2.46	CG	Right side	104.13	5.36	103.33	1.65

Covariate values in the model were appraised at the following values: left pre-test = 102.34°, right pre-test = 103.47°. A * denotes a significant difference relative to the adjusted CG group on the left side (P = 0.004); a # signifies a significant difference relative to the adjusted CG group on the right side (P = 0.001); a Δ indicates a significant difference relative to the adjusted CG group on the left side (P = 0.011); a ※ denotes a significant difference compared to the adjusted CG group on the right side (P = 0.002).

## Discussion

The hamstring muscles, located at the rear of the thigh, originate from the ischial tuberosity and insert into the tibia. Encompassing the hip and knee joints, they are predominantly composed of the semitendinosus, semimembranosus, and biceps femoris muscles. The primary functions of these muscles involve knee flexion and hip extension. They also play an important role in maintaining knee joint stability and effectively mitigating injuries to the hamstring tendons, knee, buttocks, and ankles. Dance students require good flexibility and strength in the hamstring muscle group to ensure optimal dynamic lower extremity performance.

### Effects of eccentric training on hamstring flexibility

The main factors restricting joint mobility include muscles, fascia, ligaments, and tendons^16,17^. Stretching and eccentric training augment the elasticity and plasticity of connective tissues, thereby enhancing joint range of motion^18^. Research has indicated that the maximum force produced by eccentric muscle contractions is about 50% higher than that of concentric contractions and about 25% higher than that of isometric contractions^19^. The NHE&SLD group engaged in dynamic resistance stretching, while the FBE&SLL group underwent static stretching. The distinction lies in the presence or absence of resistance. In the process of resistance, the increase in strength is the result of multiple physiological changes in its ultrafibre structure that are elongated, restored and regenerated in the resistance load. Furthermore, under intense resistance stretching, the degree of muscle elongation may be more significant. Therefore, the NHE&SLD group showed a better change in hip mobility. The related article proposed that centrifugal training has a similar increase in ROM with stretching training. Eccentric contractions can stretch muscle abdomen and tendon under higher resistance or load, providing a greater stimulus for ROM enhancement. Eccentric training facilitates changes in joint mobility by increasing the active activation of agonist muscles and reducing the co-activation level of antagonist muscles^20–22^. It stimulates voluntary activation of agonists and reduces the co-activation of antagonists to promote ROM changes^23^.The FBE&SLL group also provided some stimulation to the hamstring muscles and improved hamstring flexibility, but the stimulus load on the hamstring muscles was not as high as in the NHE&SLD group, resulting in relatively poorer improvement in hamstring flexibility. The CG group did not receive any strength intervention training specifically targeting the hamstring muscles, so there was no significant improvement in flexibility.

### Effects of eccentric training on hamstring muscle strength levels

For dance students, possessing good muscle strength is crucial for enhancing their performance and preventing injuries^24,25^. The daily training and performances of dance students involve multidimensional and multi-form posture changes, as well as comprehensive movement states such as walking, running, and jumping^26–28^. Techniques such as high leg lifts, jumps, and flips all necessitate substantial muscle strength^29–31^. Following intervention training, the NHE&SLD group demonstrated significant improvements in both concentric and eccentric muscle strength levels in the dominant leg, with a particular increase in eccentric muscle strength. There were no significant modifications in the levels of concentric and eccentric muscle strength in the FBE&SLL and CG groups. This is likely because in the NHE&SLD group's eccentric training, the muscles underwent resistance stretching whilst sustaining substantial contractile tension to counteract the body’s weight, thereby considerably enhancing muscle strength. Early studies on animal muscles^32^ have indicated that eccentric training provides an essential mechanical stimulus for amplifying the number of rat muscle fibers after downhill running^33–36^. This increase in the number of serially connected muscle fibers encourages faster contraction speed and increased muscle strength during muscle contraction^37,38^. Chronic eccentric training appears to be associated with modifications in the length-tension curve of muscles^39,40^. Research involving humans has shown that eccentric training significantly stimulates the longitudinal growth of muscle fibers. For instance, a 14% increase in the length of quadriceps muscle fibers was observed after 10 weeks of isokinetic eccentric training at the knee joint^41,42^. The FBE&SLL group's stretching training, consisting of simple static stretching without resistance, elongated the muscles and enhanced flexibility, but it did not augment muscle strength. The CG group only completed the basic training content of the course, and because there was no special strength training, there was no significant change in the strength performance area.

## Conclusion

Eccentric training can better enhance the flexibility and strength of hamstring muscles in dance students. Traditional stretching training significantly improves the flexibility of the hamstring muscles. Eccentric training has more training benefits than traditional stretching training. It is recommended for dance students to use eccentric training when increasing hamstring flexibility and strength.

## Limitations and future directions

The eccentric training exercises developed in this research are single-function movements and do not possess specific characteristics pertinent to dance. In future related studies, consideration can be given to designing eccentric training movements specifically relating to dance. The body positions for eccentric training are not confined to the hamstring muscles and can be tailored for other positions depending on the requirements of dance performances.

## Data Availability

The data that support the findings of this study are available from [Laboratory of Biomechanics of Hebei Normal University] but restrictions apply to the availability of these data, which were used under license for the current study, and so are not publicly available. Data are however available from the corresponding author upon reasonable request and with permission of [Laboratory of Biomechanics of Hebei Normal University].
